# Corticotropin-releasing hormone stimulates expression of leptin, 11beta-HSD2 and syncytin-1 in primary human trophoblasts

**DOI:** 10.1186/1477-7827-10-80

**Published:** 2012-09-12

**Authors:** Fabian B Fahlbusch, Matthias Ruebner, Gudrun Volkert, Ramona Offergeld, Andrea Hartner, Carlos Menendez-Castro, Reiner Strick, Manfred Rauh, Wolfgang Rascher, Jörg Dötsch

**Affiliations:** 1 Department of Pediatrics and Adolescent Medicine, University of Erlangen-Nürnberg, Erlangen, Germany; 2 Department of Gynecology and Obstetrics, University of Erlangen-Nürnberg, Erlangen, Germany; 3 Childrens’ and Adolescents’ Hospital, University of Cologne, Cologne, Germany

**Keywords:** CRH, leptin, 11beta-HSD2, Syncytin-1, Trophoblast, Syncytiotrophoblast, Placenta

## Abstract

**Background:**

The placental syncytiotrophoblast is the major source of maternal plasma corticotropin-releasing hormone (CRH) in the second half of pregnancy. Placental CRH exerts multiple functions in the maternal organism: It induces the adrenal secretion of cortisol via the stimulation of adrenocorticotropic hormone, regulates the timing of birth via its actions in the myometrium and inhibits the invasion of extravillous trophoblast cells in vitro. However, the auto- and paracrine actions of CRH on the syncytiotrophoblast itself are unknown. Intrauterine growth restriction (IUGR) is accompanied by an increase in placental CRH, which could be of pathophysiological relevance for the dysregulation in syncytialisation seen in IUGR placentas.

**Methods:**

We aimed to determine the effect of CRH on isolated primary trophoblastic cells in vitro. After CRH stimulation the trophoblast syncytialisation rate was monitored via syncytin-1 gene expression and beta-hCG (beta-human chorionic gonadotropine) ELISA in culture supernatant. The expression of the IUGR marker genes leptin and 11beta-hydroxysteroid dehydrogenase 2 (11beta-HSD2) was measured continuously over a period of 72 h. We hypothesized that CRH might attenuate syncytialisation, induce leptin, and reduce 11beta-HSD2 expression in primary villous trophoblasts, which are known features of IUGR.

**Results:**

CRH did not influence the differentiation of isolated trophoblasts into functional syncytium as determined by beta-hCG secretion, albeit inducing syncytin-1 expression. Following syncytialisation, CRH treatment significantly increased leptin and 11beta-HSD2 expression, as well as leptin secretion into culture supernatant after 48 h.

**Conclusion:**

The relevance of CRH for placental physiology is underlined by the present in vitro study. The induction of leptin and 11beta-HSD2 in the syncytiotrophoblast by CRH might promote fetal nutrient supply and placental corticosteroid metabolism in the phase before labour induction.

## Background

As part of the neuroendocrine system, the hypothalamo-pituitary-adrenal axis controls a wide range of body functions in humans. Hypothalamic corticotropin-releasing hormone (CRH) acts via its two receptors CRH-R1 and CRH-R2 to control stress reaction, autonomic functions, behavioural response, appetite, metabolism and the immune system.

Since its discovery in placental extracts in 1982 [1] it has become evident, that CRH and the related peptide urocortin [2] also exert important functional roles in human reproductive physiology [3,4]. CRH and its receptors are present in ovaries [5], endometrium [6], decidua [7], myometrium [8] and in the placenta (syncytiotrophoblast, chorion and amnion) [9,10]. The placental syncytiotrophoblast is a major source of plasma CRH in the maternal circulation in the second half of pregnancy [11]. Multiple isoforms of the CRH receptors CRH-R1 and CRH-R2 were identified in the placental trophoblast [9,10] and myometrium [12,13] throughout gestation. Hence differential local effects on fetal and maternal intra-uterine tissues are conceivable. The effects of placental CRH have been intensively studied indirectly via the action of the cortisol proxys leptin and 11β-HSD2 [14,15] and directly at the placental bed, where it plays an important role in the timing of birth in humans [16]. CRH interacts with progesterone to enhance the contractile response of the myometrium [16,17] and regulates the vascular tonus in the fetoplacental circulation through the nitric oxide (NO)/cGMP pathway [18,19]. Bamberger et al. [20] have recently shown, that CRH inhibits extravillous trophoblast (EVT) invasion by decreasing the expression of CEACAM1 via signalling through CRH-R1. There is growing evidence that a dysregulation of spiral artery invasion by EVT in the first trimester is a process contributing to the vascular resistance observed in the pregnancy complications preeclampsia and intrauterine growth restriction (IUGR) in late pregnancy [21,22]. In line with this finding, we and others have previously shown that placental CRH expression and CRH in maternal plasma are significantly elevated in IUGR [23-26]. IUGR is further pathophysiologically characterized by a reduction in trophoblastic syncytialisation rate [27], increased leptin [28] and reduced 11β-HSD2 [29] expression.

Although it is known that the syncytiotrophoblast is a major source of CRH in the second half of pregnancy, the role of CRH on the process of cytotrophoblastic syncytialisation and on endocrine hormone regulation in the syncytiotrophoblast is unknown so far.

To further investigate local actions of CRH on trophoblast function, we sought to determine its influence on the syncytialisation of isolated primary villous trophoblastic cells and on the expression of leptin and 11β-HSD2. We found that CRH induced leptin and 11β-HSD2 expression, without affecting syncytialisation of trophoblastic cells.

## Methods

### Placental collection and tissue culture

Six term placentas from women with singleton uncomplicated pregnancies were collected immediately after placental delivery. Elective caesarean section delivery was performed and birth weight was >10^th^ percentile according to Voigt et al. [30]. Primary human cytotrophoblasts were isolated from the placentas using the established trypsine-DNAse-dispase/percoll method as initially described by Kliman et al. [31], with additional previously published modifications [32,33]. The purity of trophoblastic cells was routinely controled by multiple FACS analysis (FACSCalibur, BD Biosciences), as described previously [33], providing at least 90% cytotrophoblasts. In short, we determined that 10–13.3% of the fractions were HLA-A,B,C + (mainly mononuclear blood cells and fibroblasts) and 86.6–90% HLA-A,B,C negative. Additionally, fractionated cells were 2.4–4.5% CD45+ (mononuclear blood cells) and 95.5–97.6% CK7+ (epithelial marker). Antibodies used: CK7/PE (clone 5 F282), Santa Cruz Bio., Heidelberg, Germany (1:20); HLA-A,B,C/PE (cloneW6/32), Biolegend, Uithoorn, Netherlands (1:10); CD45/FITC, Miltenyi Biotec, Berg. Gladbach, Germany (1:10). Hence 86.6–90% of the fractionated cells were trophoblastic cells and 10–13.3% non-trophoblastic cells. Multinucleated fractured syncytial fragments were identified via their DNA-content using propidium iodide staining (Sigma–Aldrich Chemie, Munich, Germany; 50 μg/ml), specific for DNA content. All fractured syncytial cellular fragments, non-adherent cells and debris were removed initially after 4 h and then every 24 h with a medium change [34]. Cells were subsequently seeded into 6-well Falcon plates (Becton Dickinson, Heidelberg, Germany) at a density of 3x10^5^ cells/cm^2^ and maintained in Earl’s medium 199 (PAA Laboratories, Linz, Austria) supplemented with 10% fetal calf serum (PAA Laboratories), 20 mM Hepes (Sigma–Aldrich), 0.5 mM L-glutamine (Gibco Invitrogen, Karlsruhe, Germany), penicillin (10 U/ml), streptomycin (10 mg/ml), and fungizone (0.25 mg/ml) (Sigma–Aldrich, Gibco Invitrogen, respectively). Cultures were grown at 37 °C under normoxia with 95% air, 5% CO_2_ in a humidified atmosphere using a Forma Scientific incubator (Fisher Scientific, Schwerte, Germany) as described in detail previously [33]. After incubation for 24 h, trophoblastic cells were stimulated with 0.5, 1.0 and 2.0 μg/ml (equivalent to 100, 200, 400 nM, respectively) CRH (Bachem, Weil am Rhein, Germany) for 6, 12, 24, 48 and 72 h. This range of concentrations was chosen, as it has been described to exert biological effects on trophoblasts [20]. Our pilot study showed no difference of repetitive stimulation vs. single application of CRH to the cell culture, with significant changes in gene expression between vehicle and CHR-treated groups starting with 48 h (1.0 and 2.0 μg/ml CRH) for the analysed genes. 72 h were chosen as the maximum observational period, because our previous experiments have shown that cytotrophoblast viablitiy steadily decreases afterwards. Hence for illustration of group differences the time-points of 48 h and 72 h and CRH concentrations of 1.0 μg/ml and 2.0 μ/ml are displayed in the results section only. Cultured trophoblasts as well as culture supernatants were collected at the time-points described above, snap frozen and stored at −80°C until further processing. All experiments were assayed in triplicate and were repeated using cells from different placentas.

### Ethics

The study was reviewed and approved by the Ethics Committee of the Medical Faculty of the University of Erlangen-Nürnberg (#2625 02/28/02). Written informed consent was obtained from all subjects.

### RNA isolation and reverse transcription-polymerase chain reaction (RT-PCR)

Total RNA was isolated from primary human trophoblasts using TRIzol reagent (Gibco Invitrogen) as recommended by the manufacturer. RNA was quantified by absorbance at 260 nm and the quality of RNA was confirmed using a 1% agarose gel. After DNase treatment, 1.0 mg RNA was transcribed into cDNA using M-MLV-RT (Promega, Madison, WI, USA) and Oligo dT-primer (MWG-Biotech AG, Ebersberg, Germany). DNase treatment and cDNA synthesis were carried out as previously [33,35].

### SYBR-Green based real-time PCR (2^-ΔΔCT^ – method)

As previously described [14] the mRNA expression of leptin, 11β-HSD2, Syncytin-1, CRH-R1 and CRH-R2 were quantified by normalising to the house-keeping gene hypoxanthine guanine phosphoribosyl transferase (HPRT) and confirmed with r18S as a second housekeeper, yielding the same results. Commercial reagents (Absolute Blue SYBR Green master mix, ABgene, UK) and conditions were applied according to the manufacturer’s protocol. Serial dilutions of one of the samples served as reference providing relative quantification of the unknown samples. Sequences of primers and probes are listed in Table 1. 

**Table 1:** Primer sequences

	**Forward (5´-3´)**	**Reverse (5´-3´)**
*HPRT*	CCGGCTCCGTTATGGC	GGTCATAACCTGGTTCATCATCA
*r18S*	GCAATTATTCCCCATGAACG	GGCCTCACTAAACCATCCAA
*Leptin*	ACAATTGTCACCAGGATCAATGAC	TCCAAACCGGTGACTTTCTGT
*11β-HSD2*	CATCACCGGCTGTGACTCTG	CGGCAGCCGCATGTTAG
*CRH-R1 alpha*	CTACATGCTGTTCTTCGTCAATCC	GGCAGAACGGACCTGGAA
*CRH-R2*	TCCAGTACAGGAAGGCAGTGAA	GGAGTTGAAATAGATGAACATGATCTG
*Syncytin-1*	ATGGAGCCCAAGATGCAG	AGATCGTGGGCTAGCAG

### Determination of β-hCG, leptin and LDH concentration in the supernatant

The concentration of β-human chorionic gonadotropine (β-hCG) in the supernatants of trophoblastic cells was determined by the use of an UniCel DxI 600 Access Immunoassay System (Beckman Coulter, Krefeld, Germany). The concentration of leptin in the supernatants of trophoblastic cells was determined by the RayBio® Human Leptin ELISA Kit (RayBiotech, Norcross, GA, USA) according to the manufacturer´s instructions. Lactate dehydrogenase (LDH) concentrations were obtained spectrophotometrically [36] by the In Vitro Toxicology Assay Kit Lactate Dehydrogenase based (TOX-7, Sigma–Aldrich). Determination of culture supernatant protein content for protein normalisation was performed with Pierce bicinchoninic acid (BCA) Protein Assay Reagent (Thermo Fisher Scientific, Bonn, Germany). All measurements were assayed in triplicate. Analysis of the results was performed using Ascent Software v2.6 for Multiscan photometer (Thermo Fisher Scientific).

### Statistical analysis

Results were expressed as mean ± standard error of the mean (SEM). Differences were assessed using the non-parametric Mann–Whitney *U* test provided with SPSS statistic software (v19.001, IBM, Ehningen, Germany). A p-value of <0.05 was considered significant.

## Results

### Assessment of primary trophoblastic cell viability and functionality

**Figure 1:**
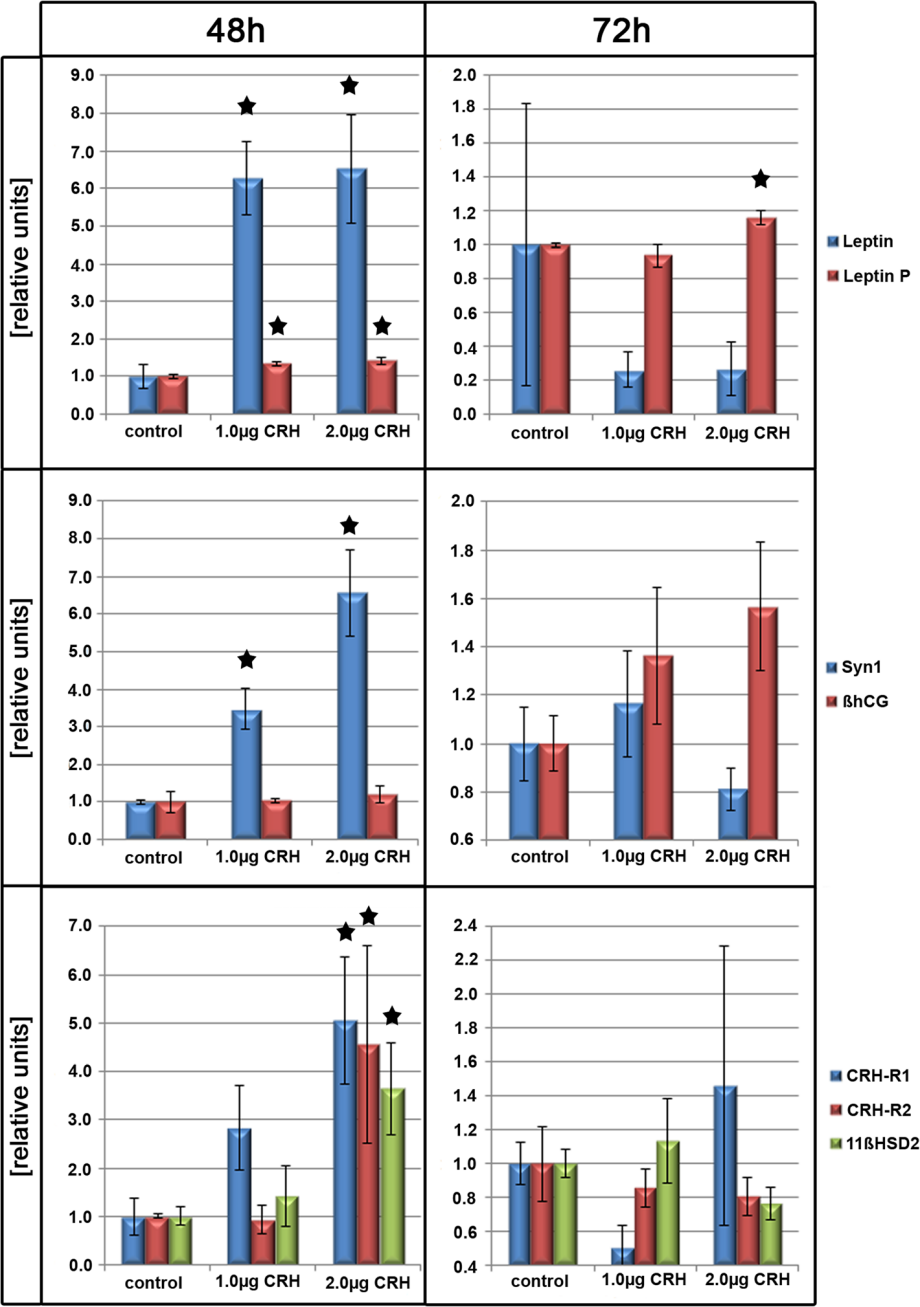
**Overview of gene expression profiles and results of protein detection.** Overview of gene expression profiles (RT-PCR) and results of protein detection (ELISA) in cell culture supernatant of vehicle and corticotropin-releasing hormone (CRH) (1.0 and 2.0 μg/ml) treated trophoblasts at 48 and 72 h**. Top row**: Leptin gene expression (Leptin, blue bars), Leptin protein secretion (Leptin P, red bars). **Middle row:** Syncytin-1 (Syn1) (blue bars), β-hCG (red bars). **Bottom row:** CRH-R1 (blue bars), CRH-R2 (red bars), 11β-HSD2 (green bars). Displayed are values relative to the control value at the designated time-point as mean ± SEM, * = p < 0.05.

At the sequential experimental time-points LDH was assessed spectrophotometrically using the trophoblastic cell supernatants. There was no significant increase observed during trophoblastic cell culture, nor were group differences detected between unstimulated controls and CRH-treated trophoblast cells in terms of viability (Table 2), ruling out a contamination of the supernatant with intracellular β-hCG as a consequence of cell lysis. The β-hCG secretion is a valid parameter of trophoblast syncytialisation rate [31]. The β-hCG content of the trophoblastic cell supernatant, as assessed by ELISA, increased continuously over the time-points investigated in both experimental groups. After 24 h of culture, the increase became significant (p < 0.01) evidencing the progression of syncytialisation of the trophoblastic cells (data not shown). Compared to 6 h, both vehicle and CRH treated primary trophoblastic cells showed a significant increase of β-hCG protein content in the supernatant at 48 h (p < 0.01) and more significantly at 72 h (p < 0.001, data not shown). Stimulation with CRH (1.0 and 2.0 μg/ml) did not influence the amount of β-hCG in the supernatant at 48 and 72 h (Figure 1), indicating that CRH does not alter maturation of trophoblastic cells in vitro. Additionally we measured syncytin-1 (Syn1) expression, as previously described [33,35]. Syn1 is essential for mediating trophoblast cell fusion events [37]. Syn1 expression was significantly induced at 48 h by CRH in a dose-dependent manner (1.0 μg < 2.0 μg, p < 0.029 for both, Figure 1). At 72 h the stimulative effect of CRH on Syn1 expression had subsided (Figure 1). 

**Table 2:** LDH absorbance in the culture medium of human trophoblastic cells with and without CRH (1 μg/ml) stimulation

**Time (hours)**	**Absorbance levels (rel. units)**				**p-value**
	**Vehicle**		**CRH**		
	**mean**	**SEM**	**mean**	**SEM**	
*6*	0.021	0.006	0.022	0.013	ns
*12*	0.036	0.005	0.032	0.001	ns
*24*	0.026	0.017	−0.004	0.003	ns
*48*	0.033	0.004	0.019	0.001	ns
*72*	0.025	0.005	0.029	0.006	ns

### Leptin expression

**Figure 2:**
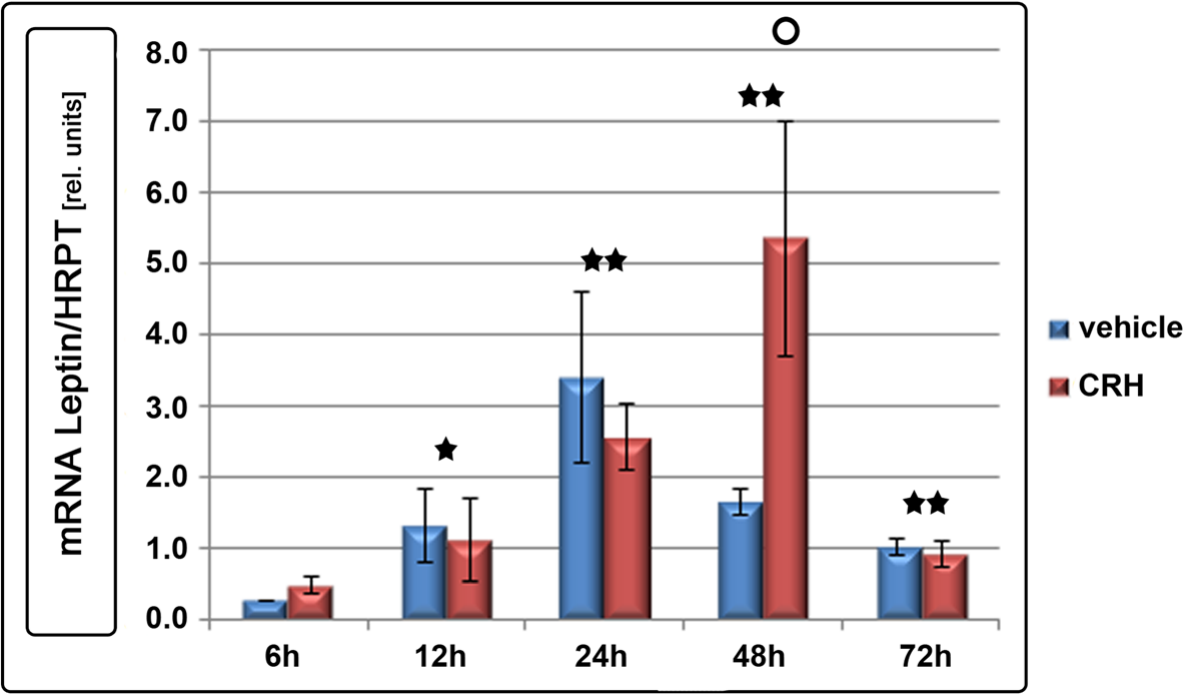
**Leptin expression in human trophoblastic cells: Vehicle vs. CRH (1.0 μg/ml) stimulation.** A significant (p < 0.05) increase in relative leptin gene expression was observed after 12 h in both groups. CRH treatment significantly (p < 0.05, circle) increased leptin expression above control levels after 48 h. Gene expression is related to the housekeeping gene HPRT. Displayed are means ± SEM, *p < 0.05, **p < 0.01.

Previous experiments have shown a close relation of trophoblast leptin expression to leptin secretion [14]. Leptin expression increased with culture time of trophoblastic cells, irrespective of the stimulation with CRH. This increase was significant after 12 h of culture and peaked after 24 h (Figure 2; p < 0.05). While leptin expression levels in the unstimulated control group declined after 48 h, CRH-treated primary trophoblastic cells showed a more sustained induction of leptin expression (Figure 2). At this time-point, leptin expression was significantly higher in the trophoblastic cells after stimulation with 1.0 and 2.0 μg/ml CRH compared to unstimulated controls (p < 0.05, Figure 1 and Figure 2). After 72 h the leptin expression had returned to a basal level in both groups alike. Leptin protein expression closely matches expressional changes following CRH stimulation (Figure 1). At 48 h CRH dose-dependently increased in leptin secretion into trophoblast culture supernatant. The stimulatory effect of 1.0 μg/ml CRH on trophoblast leptin secretion lasted for 48 h (p < 0.029). After 2.0 μg/ml CRH stimulation, there was still a significant (p < 0.002) induction in leptin secretion detectable at 72 h (Figure 1). 

### 11β-HSD2 expression

Stimulation of trophoblastic cells with 2.0 μg/ml CRH significantly (p < 0.029) induced 11ß-HSD-2 expression at 48 h (Figure 1). Stimulation of trophoblastic cells with 1.0 μg/ml CRH did not significantly alter 11ß-HSD-2 expression during the observational period (Figure 1). At 72 h the stimulatory effect of CRH had subsided for both 1.0 and 2.0 μg/ml CRH concentrations.

### CRH receptor expression

CRH-R2 gene expression was low (C_t_: 32.8 ± 0.35 SEM) in cultured human trophoblasts throughout the duration of the experiment, while CRH-R1 was readily detectable (C_t_: 24.89 ± 0.21 SEM). CRH treatment at a concentration of 2.0 μg/ml resulted in an increased CRH-R1 and CRH-R2 expression in these cells (5.05 ± 1.33 SEM, p = 0.029; 4.55 ± 2.05 SEM, p = 0.029, respectively). Interestingly, at 72 h CRH-R1 and CRH-R2 expression levels following CRH treatment (1.0 and 2.0 μg/ml) were not different to expression levels in vehicle treated controls.

## Discussion

In the present study, we aimed to clarify the influence of corticotropin-releasing hormone (CRH) exposure on the syncytialisation rate of isolated primary villous trophoblastic cells. Moreover, we investigated whether CRH induces alterations in gene expression of specific endocrine placental regulators in vitro. Our results showed a significantly higher leptin expression in trophoblastic cells, which concomitantly resulted in a significant induction of leptin protein secretion into the supernatant after 48 h of CRH stimulation, compared to unstimulated control cells. Moreover, 11β-HSD2 expression was dose-dependently induced by 2.0 μg/ml CRH after 48 h. Formation of a functional syncytiotrophoblast occurred after 24 h in the CRH-stimulated and the control group to the same degree, as determined by increasing β-hCG secretion without a concomitant increase of cell lysis reflected by constant LDH levels in the supernatant throughout the experiment [31]. Syncytin-1 expression, a key regulator gene of trophoblast syncytialisation [33,35,37], was dose-dependently induced after 48 h of CRH stimulation only.

The finding that leptin expression increases with the progression of trophoblast syncytialisation in both groups is in line with results from Ashworth et al. [38], who showed that placental leptin expression is an exclusive feature of the syncytiotrophoblast (SCT), with a reduced leptin expression in undifferentiated cytotrophoblasts. Likewise, CRH was described as a syncytical peptide [39]. CRH-treated and vehicle stimulated trophoblasts showed an equal increase of ß-hCG concentration in the culture supernatant, while cell lysis was low and not different (as determined by LDH). This finding is indicative that the rate of trophoblast syncytialisation gradually increased over time in both groups [40]. The induction of leptin expression after 24 h of culture of CRH- and vehicle treated trophoblastic cells can therefore be attributed to trophoblastic differentiation into functional syncytium. The significant difference in leptin gene expression in CRH-treated and vehicle treated control groups at 48 h and the induction of leptin protein secretion into the cell culture supernatant, however, do not seem to be solely an indirect effect of syncytialisation, as ß-hCG levels of CRH-treated trophoblastic cells were not significantly different from the ß-hCG levels of vehicle treated controls. Hence the significant increase in leptin expression and secretion of stimulated cells at 48 h rather seems to be a direct effect of CRH, possibly via activation of CRH-R1α, which was readily detectable in trophoblasts [10,41] and towards which CRH shows a ten times higher affinity as compared to CRH-R2 [42], whose expression was significantly lower. The fact that we were able to detect CRH-R1α and CRH-R2 in vitro does not rule out the possibility of CRH signalling via its other known placental isoforms that were described in vivo [10,41]. Interestingly Karteris et al. found significant levels of receptor hybridization foremost in syncytiotrophoblast and to a lesser extend in scattered cytotrophoblasts, which is supported by almost exclusive binding of ^125^I]CRH to purified syncytiotrophoblast [43]. This could suggest CRH might rather excert its functions after spontaneous syncytialisation of cytotrophoblasts in vitro than on single cytotrophoblasts.

To address the functional relevance of our findings studies with CRH antagonists, such as CRH antagonists antalarmin (CRH-R1) and antisauvagine (CRH-R2) would be needed.

We found that CRH (2 μg/ml) dose-dependently induced CRH -R1α and –R2 gene expression at 48 h (Figure 1, p < 0.029). The stimulatory effect of CRH on leptin expression was abolished at 72 h. CRH-R internalization could be a possible mechanism to explain this phenomenon, as previously described by others [44,45]. CRH-R internalization could also account for the absence of an expressional effect on the studied target genes following repeated CRH stimulation in our pilot study. Such an induction of CRH-R expression levels following exposure to higher doses of CRH might optimize CRH signal transduction. Interestingly, higher concentrations of CRH (2.0 μg/ml) also significantly induced 11ß-HSD2 expression at 48 h (Figure 1, p < 0.029), while 1.0 μg/ml showed no such effect. Hence higher levels of auto-/paracrine CRH might hypothetically prepare the syncytium to cope with a CRH-triggered increase of maternal cortisol more efficiently.

The above findings regarding leptin are in line with findings from our previous experiments showing a close relationship of trophoblast leptin expression and secretion rate in vitro following dexamethasone stimulation [14]. Interestingly, dexamethasone stimulation (10 μM) produced a more pronounced leptin secretion (~120 pg/ml at 72 h) when compared to CRH (1.0 and 2.0 μg/ml) stimulation (~56-61 pg/ml at 72 h, respectively). However, dexamethasone clearly induced ß-hCG secretion. Hence in contrast to CRH the effect of dexamethasone on leptin secretion seems to be partly attributable to an increased rate of trophoblast differentiation and maturation, as also seen by Audette et al. [46]. They were also able to demonstrate a trend to Syn1 induction in placental explants following dexamethasone treatment.

The fact, that we found an increase in Syn1 expression following CRH stimulation without a concomitant increase in ß-hCG might point to a differential regulation of the two genes. A common pathway for both Syn1 and ß-hCG stimulation is the forskolin triggered induction of cAMP [47]: An activation of adenyl cyclase (AC) raises intracellular cAMP levels and leads to PKA activation via interactions with AKAPs and downstream phosphorylation of p38MAPK and ERK1/2. Accordingly, CRH was found to induce cAMP in human endometrium via CRHR1 triggered protein-kinase A (PKA) [48] and we recently found that Syn1 is induced via the cAMP pathway in endometrial carcinoma [49]. However, as ß-hCG was not induced, CRH might either use alternative signalling pathways, such as signalling via PKC [45,50], or the detected Syn1 expression might come from a source other than the cytotrophoblast. However our cytotrophoblast isolation is ~90% pure. Thus, we cannot exclude possible minor fractions of extravillous trophoblasts (EVT) in our cell culture. EVT express CRHR1 [51] and show Syn1 expression [20,52]. Therefore, we cannot completely rule out that the Syn1 induction measured is derived from EVT. But due to their extremely small fraction in the isolation, a six-fold increase in Syn1 expression by EVT following CRH-stimulation seems rather unlikely. Another explanation could be, that CRH fosters maintenance fusion events instead of functional fusion processes, that would be reflected in ß-hCG secretion. Importantly, Syn1 is related to trophoblast processes beyond its fusogenic nature. Possible functions of the syncytin proteins are suppression of the maternal immune response against the developing fetus [53] and induction of placental immunity against vertical transmission of retroviral infections [54]. We observed a stimulatory effect of CRH (2.0 μg/ml after 48 h) on the expression of 11ß-HSD2 in primary cultured cytotrophoblasts. Like leptin, the induction of 11β-HSD2 by CRH subsided at 72 h, possibly due to CRH-receptor internalization, as discussed above. In a previous study using the same in vitro setup, we were able to show that dexamethasone (10 μM) similarly stimulates both leptin and 11β-HSD2 expression in primary trophoblastic cells [14]. 11ß–HSD2 gene expression in human placental trophoblasts grown in primary culture has been shown to maintain the same pattern as in vivo [55] and dexamethasone stimulation regularly results in an increase in 11ß–HSD2 protein expression in trophoblasts [56]. Upon the finding that CRH induces 11β-HSD2 expression one cannot draw conclusions about the activity of placental glucocorticoid metabolism. Interestingly, Friedberg et al. [57] found a CRH-induced reduction of 11ß-HSD1 activity in human adipocytes in vitro. In isolated cytotrophoblasts Sharma et al. [58] were unable to induce 11ß-HSD2 activity using CRH concentrations of 1-100 ng/ml, however, they were able to identify the CRH downstream signalling protein p38MAPK [47] as an essential regulator for 11ß-HSD2 activity. The fact, that we observed expressional changes of 11β-HSD2 following CRH treatment at much higher CRH dosages (2.0 μg/ml) could however imply a possibility of a CRH-driven glucocorticoid induced feed-forward mechanism on 11ß-HSD2 activity. Although such a mechanism has not been described for the placenta yet, the subsequent reduction of cortisol availability might be an intriguing regulatory function shielding the fetus of placental CRH-induced maternal glucocorticoids.

Our study focused on the auto- and paracrine effects of CRH on leptin production in isolated trophoblasts, as the placenta co-expresses both leptin (ObR-L) [59] and CRH receptors [9]. We were able to show a significant increase in leptin expression in syncytialised trophoblastic cells following CRH treatment. While the exact auto- and paracrine mechanisms and the functional role of the interaction of CRH and leptin at the level of the syncytiotrophoblast remain to be determined, an increase of endocrine CRH and leptin expression might translate into endocrine signals affecting both fetus and mother, besides their local influence on the trophoblast.

In this respect it is noteworthy, that the major fraction of placental leptin and CRH is secreted into the maternal circulation [60,61]. Nevertheless, the syncytiotrophoblast is also involved in the maintenance of fetal leptin and CRH serum levels [62,63]. Besides its role in fetal organ maturation via cortisol induction, there is in fact evidence, that placental CRH drives parturition via induction of adrenal DHEA-S on the fetal side followed by an increase in placental estrogen secretion [63].

In IUGR, a condition characterised by increased fetal serum CRH levels [25], we found unchanged leptin levels in fetal umbilical cord blood [23], despite an elevated placental leptin mRNA and protein expression [28,64]. Hence, it seems likely that CRH and CRH-induced leptin (as suggested by our results) might interact on the maternal side.

White et al. [65] showed that leptin has lipolytic effects in rat placental tissue in vitro. CRH antagonises lipolysis via down-regulation of 11ß-HSD1 in adipose tissue [57]. Hypothetically leptin and CRH might act together in regulating the maintenance of fetal nutrient supply at the placental level.

## Conclusions

In summary, our data indicate that CRH stimulation induces leptin secretion in the human syncytiotrophoblast in an auto-/paracrine fashion. Similarly, CRH induced 11ß-HSD2 expression. This suggests a short-loop feedback of CRH-induced leptin on CRH action at the feto-maternal interface. Such a putative cross-talk could play an essential role in the regulation of syncytiotrophoblast nutrient supply and cortisol metabolism, besides possible further implications for myometrial contractility, placental bed perfusion and the timing of birth. Furthermore CRH-induced 11ß-HSD2 might locally determine placental corticosteroid metabolism and thereby the passage of placental CRH-triggered maternal cortisol via the syncytium to the fetus. This would protect the fetus from detrimental elevated maternal glucocorticoid exposure. The underlying mechanism and the functional role of the interaction of CRH with leptin and 11ß-HSD2 at the syncytiotrophoblast remain to be determined.

## Competing interests

The authors declare that they have no competing interests.

## Authors’ contributions

FBF contributed to conception and design of the study, analysed and interpreted the data and drafted the manuscript. MR performed the cell culture experiments, including RT-PCRs. GV performed ELISA analysis. RO contributed to acquisition and analysis of the data, AH contributed to interpretation of data and was involved in drafting the manuscript, MR and CM-C were involved in the analysis of data and critically revised the manuscript for important intellectual content, RS contributed to the acquisition and analysis of data, WR critically revised the manuscript for important intellectual content, JD contributed to the conception and design of the study and critically revised the manuscript for important intellectual content. All authors have given the final approval of the version to be published.
